# Two Transcutaneous Stimulation Techniques in Shoulder Pain: Transcutaneous Pulsed Radiofrequency (TPRF) versus Transcutaneous Electrical Nerve Stimulation (TENS): A Comparative Pilot Study

**DOI:** 10.1155/2019/2823401

**Published:** 2019-02-04

**Authors:** Mu-Lien Lin, Hung-Wei Chiu, Zao-Ming Shih, Po-Ying Lee, Pei-Zhi Li, Chin-Hong Guo, Yuan-Jie Luo, Shen-Chieh Lin, Kwan-Yu Lin, Yu-Ming Hsu, Angela Pang, Weiwu Pang

**Affiliations:** ^1^Department of Anesthesiology, Assistant Professor, National Yang-Ming University, Taipei, Taiwan; ^2^Department of Anesthesiology, Taipei City Hospital, Zhongxing Branch, Taipei, Taiwan; ^3^Department of Electronic Engineering, Associate Professor, National Taipei University of Technology, Taipei, Taiwan; ^4^Feng Chia University, Taichung, Taiwan; ^5^Department of Emergency and Critical, Care Medicine, Kuang Tien General Hospital, No. 117, Shatian Road, Shalu District, Taichung City 433, Taiwan; ^6^Department of Electronic Engineering, National Taipei University of Technology, Taipei, Taiwan; ^7^Department of Surgery, Staff Physician, National Taiwan University Hospital, Taipei, Taiwan; ^8^Independent Scholar, Bachelor Degree of University of Florida in Psychology and English, 8257 Via Vivaldi, Orlando, FL, USA; ^9^Anesthesiologist, Kuang Tien General Hospital, No. 117, Shatian Road, Shalu District, Taichung City 433, Taiwan

## Abstract

**Objective:**

To compare the safety and efficacy of 2 transcutaneous stimulation techniques, transcutaneous pulsed radiofrequency (TPRF) versus transcutaneous electrical nerve stimulation (TENS), in chronic shoulder tendonitis.

**Design:**

A prospective, randomized, and double-blind clinical trial.

**Setting:**

Academic pain service of a city hospital.

**Subjects:**

Fifty patients with sonography-confirmed shoulder tendonitis.

**Methods:**

Fifty patients were randomly allocated into two groups for electrical stimulation treatment with 3-month follow-ups: Group 1 (*n*=25), TENS and Group 2 (*n*=25), TPRF. Both groups underwent either treatment for 15 minutes every other day, three times total. Our primary goals were to find any treatment comfort level, adverse event, and changes in Constant–Murley shoulder (CMS) scores. The secondary goals were finding the changes in pain, enjoyment of life, and general activity (PEG) scores.

**Results:**

For primary goals, no adverse events were noted throughout this study. No differences were found between groups for treatment tolerability (3.20 + 0.87 vs. 2.16 + 0.75). Statistically significant lower PEG scores were noticeable with the TPRF group after the course (12.73 + 5.79 vs. 24.53 + 10.21, *p*=0.013). Their statistical significance lasted for 3 months although the difference gap diminished after 1 month. CMS scores were significantly higher in the TPRF group (70.84 + 6.74 vs. 59.56 + 9.49, *p*=0.007) right after treatment course but the significance did not last.

**Conclusions:**

In treating chronic shoulder tendinitis using two transcutaneous stimulation techniques, both TPRF and TENS are safe and effective. TPRF is superior to TENS.

## 1. Introduction

Shoulder pain is a common pain complaint in practitioner consultations and has high socioeconomic cost [1, 2]. The pain may be acute or chronic in nature and originates from different anatomical sites such as joints (arthritis), capsules (adhesive capsulitis), tendons (biceps tendinitis, rotator cuff tear, rotator cuff impingement, or rotator cuff tendinitis), bursae (subacromial bursitis), or the suprascapular nerve (entrapment) [3, 4]. More recent evidence suggests that most cases of pain are nonsurgical conditions that can be confirmed by ultrasound evidence: a hypoechoic thickening indicating tendinosis [5] versus splitting indicating tear [6]. In addition to medications, steroid injection, and exercise therapy, refractory shoulder tendonitis has been treated with electrical stimulation (ES) such as transcutaneous electrical nerve stimulation (TENS), with variable success [3, 7].

Due to its noninvasive, nonmechanical, and nonpharmacological effects, TENS is one of the most common topically applied treatments to provide pain reduction, tolerance for rehabilitation, and a more active lifestyle [3, 8]; however, recent studies suggest controversies in its effectiveness compared to other more recent treatments such as transcutaneous pulsed radiofrequency (TPRF) [9–12].

One reason that TENS provides less pain relief may be related to high skin impedance that prevents ES from penetrating deeply enough to stimulate painful areas in tendonitis. This shortcoming may be overcome by the more conductive and deep-penetrating TPRF [13, 14].

Since Faraday's discovery in 1831 of a varying magnetic field which can be used to induce an electrical current, PRF driven by a voltage percutaneously has been better than conventional, continuous radiofrequency in delivering ES without raising the needle tip's temperature of the electrode beyond 42°C. This prevents neural damage on the dorsal root ganglion in patients with failed back surgery with unilateral symptomatology [15]. Transcutaneously, PRF can be modified to emit an ES using skin pads akin to that of TENS for tendonitis shoulder pain as a noninvasive technique with minimal risks [9, 10, 16].

However, controversy remains over the issue that TPRF is better than TENS in the treatment of shoulder pain [11, 12]. We therefore designed this prospective, randomized, and double-blind pilot study to compare TPRF with TENS.

## 2. Research Design and Methods

This paper analyzes the safety and efficacy of TPRF and TENS in treating tendonitis shoulder pain using transcutaneous pads. With the approval of the Taipei City Hospital Institutional Review Board, the Institutional Ethical Committee, and the informed consent of each patient, 50 adult patients with shoulder tendonitis confirmed by sonography were enrolled in this parallel, prospective, randomized, and double-blind trial at Zhongxing Branch of Taipei City Hospital, Taipei, Taiwan, between August 1, 2013 and July 31, 2014. A patient study registry was established in January 1, 2014, and all patients who were enrolled were recorded to the registry. The registration number was TCHIRB–1020523.

For homogeneity, inclusion criteria were (1) a history of shoulder discomfort for more than 3 months, (2) sonographic evidence of shoulder tendinosis (as opposed to rotator tear), (3) age between 25 and 65 years of age, and (4) use of medication and/or exercise therapy and nonopioid medications. Exclusion criteria included: (1) difficulty in communication, (2) a history of neurological, psychological disorders, or substance abuse, (3) obesity with a BMI > 30, (4) pregnancy, (5) American Society of Anesthesiology (ASA) status III or more, (6) a history of shoulder surgery, (7) high-frequency (9–17 MHz) [17] ultrasound-confirmed rotator cuff tears or calcified tendinitis, and (8) shoulder instability. All participants received a complete physical examination, X-rays, and routine lab data to rule out any disease condition that would cause shoulder pain.

## 3. Patient Instructions and Pain Assessment

Before randomization, an interview was performed by a special project physiotherapist. The patient was told that he/she would anticipate a randomly allocated ES treatment procedure using either a TPRF (self-invented, Taipei University of Technology, Taipei, Taiwan) or TENS (JS-N206B, Jian-Sen, Taipei, Taiwan) for 15 minutes every other day for a total of 3 consecutive times at the clinic. They were also told that 3 assessment questionnaires would have to be completed. One is the treatment comfort level which would be filled out by the patient at the end of the treatment course. The other two, the Constant–Murley shoulder (CMS) score [18] and the PEG (pain, enjoyment of life, and general activity) score [19], would be filled out by the patient before and after the treatment and at follow-ups one week, one month, and 3 months later.

## 4. Randomization and Blinding

**Figure 1:**
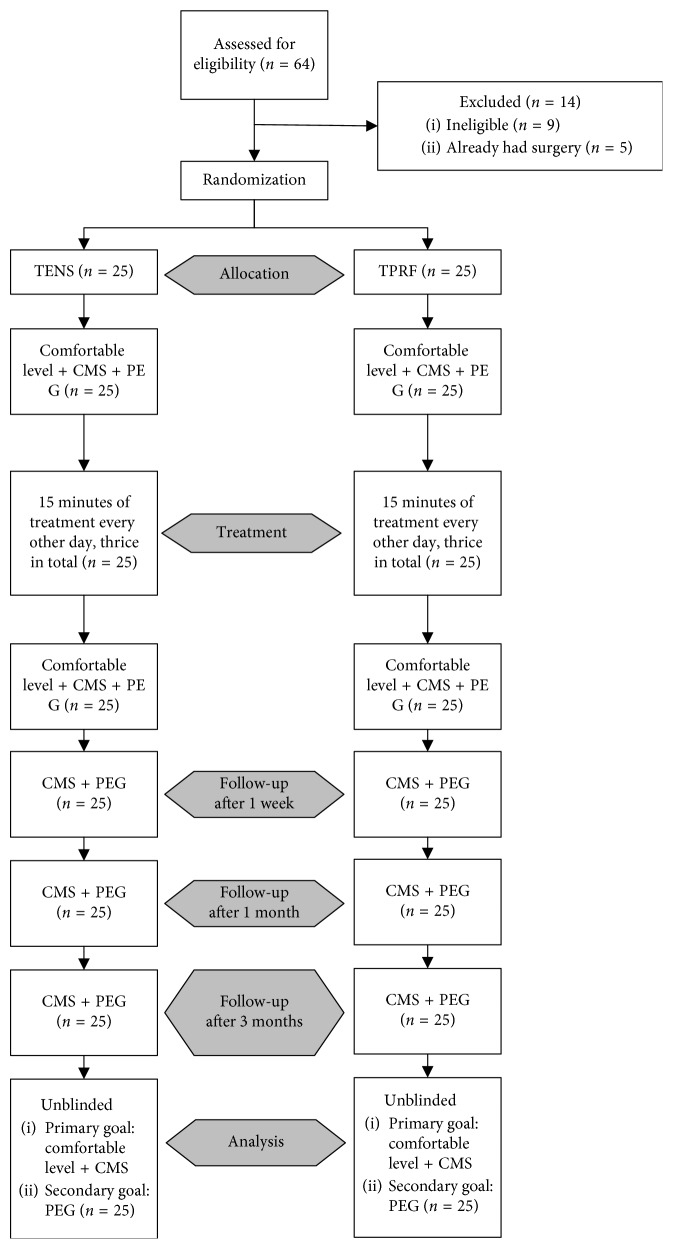
Flow diagram showing the sequence of randomization, blinding, treatments, data collection, and analysis. TENS = transcutaneous electrical nerve stimulation; TPRF = transcutaneous pulse radiofrequency; CMS = Constant–Murley Shoulder score; PEG = pain, *P* (scored by visual analog score “VAS,” scored from 0 to 100), enjoyment of life, *E* (scored from 0 to 100), and general activity, *G* (scored from 0 to 100).

Fifty patients out of the 64 recruited were enrolled chronologically into this study. After the interview, informed consent, and preprocedural safety list check, each patient received a computerized randomization grouping code (1, TENS and 2, TPRF) that was concealed in a chronologically numbered opaque envelope to randomly assign them into either Group TENS (*n*=25) or Group TPRF (*n*=25). The envelope was handed to the ES technician who did the ES treatment according to the number inside the envelope. The devices were concealed in identical cases to prevent the patient from knowing which ES treatment they received. The project physiotherapist and clinician who did not know the type of ES treatment received performed their usual managements at designated follow-ups for 3 months. The questionnaires and assessments were done by the physiotherapists. The clinical treatments were done by the clinicians. The designated follow-up times by the physiotherapist and the clinician after the ES treatment were at 1 week, 1 month, and 3 months. Thus, all clinicians and participants were double-blinded to the type of treatment (Figure 1).

## 5. Procedures

**Figure 2:**
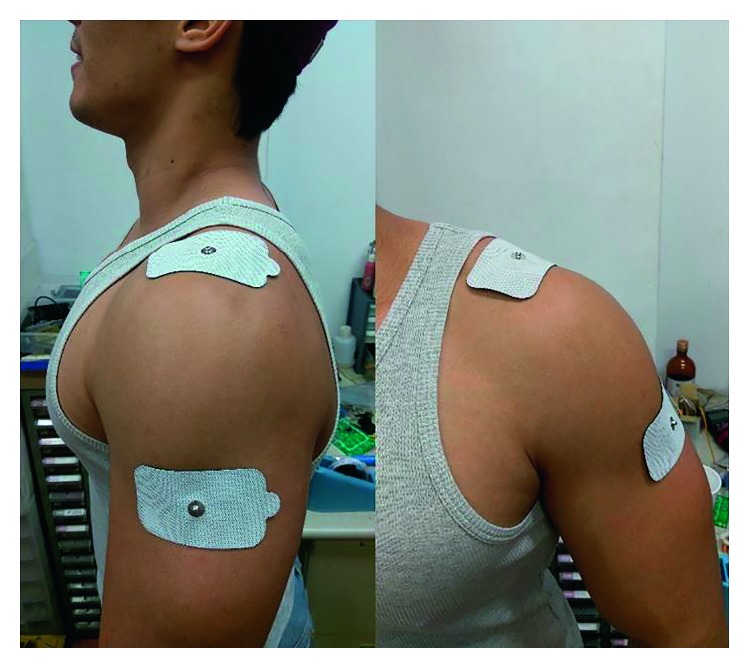
Electrical pad placement: one electrical pad (9 cm × 5 cm) attached at the maximally tender area. The other pad was attached at the inferior margin of the deltoid muscle on the same shoulder.

Before the ES treatment, the ES technician confirmed the maximally tender area for the attachment site of the first electrical pad (9 cm × 5 cm). The other pad was randomly attached close to the inferior margin of the deltoid muscle on the same shoulder (Figure 2). Then, the two leads were connected to either a TENS or a TPRF generator according to the grouping code. The ES treatment would be going for 15 minutes, every other day, three times total.

## 6. The Devices

The ES devices were set to comply with the Draft Guidance for Industry and FDA guidelines. Given Jian-Sen JS-N206B is a multiple-use electronic stimulator, the machine provides many waveform parameters to be adjusted for treatment in electroacupuncture (EA) and TENS. For TENS, the TENS waveform delivers an asymmetric triangular waveform with a pulse width of 700 *μ*s, a peak voltage of 100 volts, and high-frequency stimulation of 150 Hz. For TPRF, the PRF waveform with a voltage of 100 volts, 500 kHz pulse frequency, 2 Hz repetition rate (2 pulses per second), and 50 ms pulse duration was applied. High-frequency (HF) TENS is more comfortable for the patients [20], so as to allow patients in two different experimental groups not to feel irritation difference and prevent psychological effects. In addition, in order to reduce the interference of the use of the drug on the experimental results, it is advantageous to use HF stimulation [21].

We used 5 cm × 9 cm AXELGAARD Neurostimulation Electrodes (INDUSTRIAL WAY, FALLBROOK, CA 92028 USA) as adhesive electrodes and connected them to either the TENS or TPRF device. The settings of both the devices were managed by electrical engineers to enhance the patient's safety and comfort.

## 7. Clinical Goals

Our primary goal for safety and efficacy was to measure adverse events such as discomfort, hematoma, injury, or hyperalgia that would have occurred during this study, the treatment comfort level at the completion of the treatment course, and the changes in the CMS score before and after the treatment course, all during 1-week, 1-month, and 3-month outpatient visits. The patient treatment comfort level is a treatment satisfaction score between 0 and 5 (with 5 being the most comfortable) surveyed at the end of the ES treatment course.

The CMS score, a standardized tool for evaluating shoulder pain and function, ranges between 0 and 100 with 100 meaning asymptomatic. The score corresponds with the sum of two components, a subjective part (35 points, including 15 points for pain severity and 20 points for activity levels affecting sleep, recreation, and work) and an objective part (65 points, including 25 points for shoulder muscle strength which we assessed with an electronic dynamometer, and 40 points for range of motion without pain in forward flexion, lateral abduction, external rotation, and internal rotation). The higher the score, the better the quality of the function. The CMS score has been validated and shown good intra- and interobserver reproducibility [18].

The secondary goal was measuring the changes in PEG score, a three-item scale assessing pain intensity and its interference with emotional and physical functions. PEG stands for pain (VAS, 0 to 100, with 0 meaning no pain and 100 meaning the most pain imaginable), interruption of life enjoyment (E, 0 to 100, with 0 meaning not at all and 100 meaning complete interference), and interruption of general activity (G, 0 to 100, with 0 meaning not at all and 100 meaning complete interference). It represents the patient's subjective opinion of treatment efficacy. A low PEG score means a better quality of life. To be comparative with a 100-point CMS score scale, the average of modified *P*, *E*, and *G* scores (also 100 points instead of 10 from the literature) would be used as a validated scale for statistical analysis [19].

## 8. Sample Size Justification

As suggested by Cohen [22], conventional analytical results with a desired power level of 80% (meaning 20% Type II error) at 0.05 significance level (Type I error equivalent to a 95% confidence interval) are considered to be commonly accepted, tolerable, and low-probability mistakes. Judged by the drastic difference of treatment results of TPRF over TENS seen in the clinical trial of this work, we anticipated a large effect size (a way of quantifying significance of the difference between two group means in proportion to their standard deviation) and a customary Cohen's *d* = 0.80, indicating 25 patients in each group, as the appropriate sample size for this pilot study [23].

## 9. Statistical Analysis

We use SPSS to perform an independent *t*-test to compare the differences between Group TENS and Group TPRF that include patient demographics (age, gender, and weight), their ASA physical status, duration of illness, treatment comfort level, and treatment effectiveness (CMS and PEG scores) before and after the treatment course, at 1-week, 1-month, and 3-month follow-ups. We also used SPSS to perform an ANOVA and post hoc analysis for significant group differences.

## 10. Results

Sixty-four patients were recruited. Five had a history of shoulder surgery, 4 had sonographic evidence of rotator cuff tear, and 5 had an ASA physical status of III or more and thus were excluded. The remaining 50 were eligible and randomly assigned to either the TPRF group (*n*=25) or the TENS group (*n*=25) without protocol deviation. No patients were lost in the 3-month study period.

According to Levene's test for group equality, there were no significant group differences between the TPRF and the TENS group in age, weight, ASA physical status, duration of illness, and CMS/PEG scores before treatment except for gender (Table 1). The *t*-test about the average number of males and females within the group suggests this difference between the two groups is not significant (*p*=0.234).

## 11. Primary Endpoints

**Figure 3:**
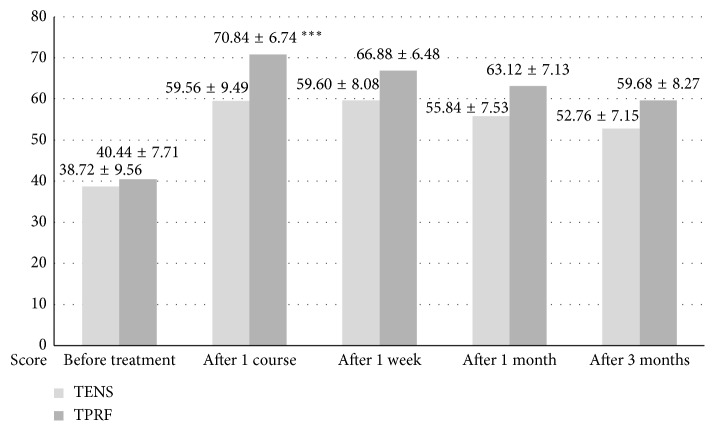
Mean CMS scores between the TPRF and the TENS group. ^*∗∗∗*^*p* ≤ 0.001.

First, there were no adverse events (such as discomfort, hematoma, injury, or hyperalgia) throughout this study. Second, patients reported being more comfortable after being treated with TPRF than TENS (3.20 + 0.87 vs. 2.16 + 0.75) although the difference was insignificant (*F*=0.601, *p*=0.442, *t*-test). Third, the CMS scores of both groups improved after the treatment course, and the TPRF group improved more than the TENS group (75% vs. 54%) which is significant clinically as defined by a 30 to 50% pain relief inferred from the IMMPACT recommendations [24–27]. There were higher CMS scores (which means more improvement) in the TPRF group at the 1-week, 1-month, and 3-month follow-ups as well. However, an independent *t*-test revealed that only the CMS difference after the treatment course (70.84 + 6.74 vs. 59.56 + 9.49, *p*=0.007) was statistically significant (Figure 3). The estimated effect size is 32.8% (meaning a strong correlation) with an observed power of 99.7% (Table 2).

## 12. Secondary Endpoints

**Figure 4:**
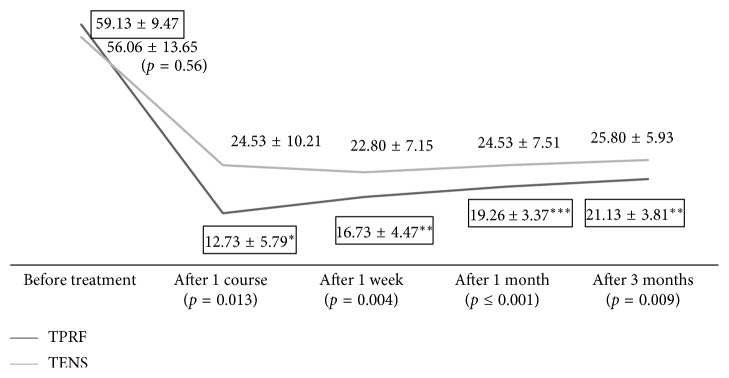
PEG score with TPRF and TENS group. ^*∗*^*p* < 0.05, ^*∗∗*^*p* < 0.01, and ^*∗∗∗*^*p* ≤ 0.001. A lower PEG is better.

Our study showed reductions in PEG scores in both the groups, 4.6-fold in the TPRF group versus 2.3-fold in the TENS group. The PEG scores between the TPRF and the TENS group are not different before treatment but are significantly different after one course of treatment, and at 1-week, 1-month, and 3-month follow-ups (Figure 4). Our study showed reductions in PEG scores in both the groups, 78% in the TPRF group versus 56% in the TENS group, significant clinically as defined by a 30 to 50% pain relief inferred from the IMMPACT recommendations [22–25]. Subsequent ANOVA (analysis of variance) tests and Scheffe's post hoc analysis for group differences revealed that this significance remained for up to 3 months but started diminishing after a month. The estimated effect size was 13% (moderate correlation) with an observed power level of 88% (Table 3).

## 13. Discussion

As evident from both results of CMS and PEG scores, our study demonstrated that both the TPRF and the TENS group showed improvement in treating chronic tendonitis shoulder pain. Before ES treatments, the two groups were statistically equal in patient demographics, ASA status, duration of illness, and CMS/PEG scores. There were no adverse events throughout the study. No difference was found between groups for tolerability during treatment. The TPRF is more effective than the TENS right after the treatment course by both CMS and PEG scores. Statistically significant lower PEG scores were noticeable with the TPRF group after the course, and the statistical significance lasted for 3 months. CMS scores were significantly higher in the TPRF group right after treatment course but statistical significance did not last.

The CMS score is a 100-point scale first introduced in 1987 and widely accepted as a reference standard for assessing shoulder function [16, 18]. The PEG score is a brief and straightforward multidimensional pain measure that could improve the initial assessment and follow-up of chronic pain [19]. Based on CMS and PEG, the TPRF is a superior ES to TENS in treating shoulder tendonitis with no noticeable complication.

The differences between the two scoring systems, CMS or PEG, are in their particular attributes. The CMS score consists of a 35% subjective portion (pain severity and activity) and a 65% objective portion (muscle strength and range of motion), whereas the PEG score is 100% subjective (pain, interruption of life enjoyment, and interruption of general activity). Both tests overlap for 35% subjectively. It can be inferred, based on the results of our study, that the participants feel significant improvement with TPRF, objectively in muscle strength and range of motion after the treatment course and subjectively in fewer interruptions in life enjoyment and general activity for up to 1 month afterward.

TENS is used by hundreds of thousands of people all over the world for the relief of physical pain. The effects of TENS have been explained by the gate control theory and are the most advanced explanation [20]. The gate control theory suggests that there is a neural mechanism in the spinal cord that acts as a kind of gate, shutting down or opening up the flow of signals from the periphery to the brain. Another theory is called the endorphin release, which suggests that electrical impulses stimulate the production of endorphins and enkephalins in the body. These natural morphine-like substances block pain messages from reaching the brain, in a similar fashion to conventional drug therapy, but without the danger of dependence or other side effects [20].

However, TPRF has better energy penetration. The impedance of the human skin (approximately 1-2 MΩ) is larger than the underlying tissues (approximately 500–1.5 KΩ) [28]. In TENS, although the ES frequency and intensity are adjustable, the frequency is very low (about 150 Hz) compared to TPRF (about 500 KHz). Due to the difference in frequency, the TENS cannot penetrate the skin as TPRF does, i.e., TPRF can deliver more energy down to the nerves and underlying tissues.

This is because that the skin and tissues can be regarded as a circuit with both impedance and capacitance paralleled. Electricity conduction depends on their impedance and capacitance, namely, capacitor impedance [29, 30].

According to the electrical theorem,

(1) $$\begin{array}{c} Z=\frac{1}{2\pi fC}, \end{array}$$

where *Z* = impedance, *f* = frequency, and *C* = capacitance of the capacitor. Frequency influences capacitor impedance in an inversely proportional way. So, the higher the stimulation frequency, the lower the capacitor impedance. Since TPRF has a much higher frequency, TPRF therefore is more conductive and has a deeper penetrating energy than TENS [13]. It also explains why the low-frequency TENS can only conduct through the skin while the high-frequency TPRF conducts through both the skin and deeper tissue and reaches more neuronal fibers, resulting in better pain relief.

In our study, the postcourse PEG scores between the TPRF and the TENS group were significantly different right after treatment, and at 1-week, 1-month, and 3-month follow-ups, but the ANOVA and post hoc analysis showed the difference between the two groups diminishes after 1 month. We can still conclude the longer, superior effects of TPRF over TENS in this treatment study. We simply believe that this is because the ES analgesic effect diminished over time in both the groups.

Other proposed hypothetical mechanisms of TPRF include heat lesioning, electric field effects, electroporation, magnetic field, and immune modulation of inflammatory cytokines [31]. It is still not clear how pulsed radiofrequency treatment works; however, we believe that available evidence suggests TPRF treatment has better energy penetration that works with a temperature-independent mechanism mediated by an ES-induced electromagnetic fields [9].

This study differs from the randomized study by Korkmaz et al. [11] that concluded no difference in effect between TENS and pulsed-radiofrequency treatment for chronic tendinosis shoulder pain in 2 aspects. First, a treatment applicator pad was placed directly over the site of maximal pain transcutaneously in this study, whereas the percutaneous pulsed radiofrequency was applied by a needle to the suprascapular nerve in Korkmaz's. It was possible that our study covered a disease-specific area rather than the suprascapular nerve per se for stimulation. Second, the TPRF group underwent a 15-min session of treatment every other day three times at 100 volts in this study, whereas there was a total of 4 minutes of treatment at 45 volts for 200 microseconds in their pulsed-radiofrequency study. The treatment energy could be different. The average current delivered to patients was measured around 200 mA.

We acknowledge these limitations: (1) a double-blind, randomized, and placebo-controlled trial is the gold standard. Our study is a novel therapy using a self-invented TPRF stimulator which can operate up to 1 kV. The study was double-blind but not placebo-controlled. However, TENS is a recognized and commonly used entity clinically with evidence-based mechanisms of action. We believe readers would have a clear image on the effects of TPRF when compared to TENS. It also seems unethical to have a controlled group that does not even have actual treatment. (2) The Cohen's chosen indicates a larger effect size. Our sample size (*n*=25 per side) is rather small for the effect size, particularly when evaluating efficacy, for both the TENS (control) and TPRF (experimental) groups' pain improved. However, our study is a pilot study on the exploration of the practicality of TPRF, serving as our basis for future studies. (3) TENS may create a tingling sensation whereas patients do not perceive any sensation with TPRF, so how were patients blinded to group allocation? None of the patients knew about which treatment they would receive and which treatment would cause tingling sensations. All they cared about was improving their shoulder pain. (4) Cost-effectiveness is an important measurement; it is not included in the study. However, the newly invented TPRF is a lot cheaper than conventional retail PRF.

TPRF is an office-based treatment that requires no sedation and is needleless, portable, noninvasive, painless, and easy to use. It provides a valuable window when an early physiotherapy is considered, being better than TENS. TPRF could potentially be used for treating other pain conditions at other locations. This study may serve as a background for future TPRF improvements, such as determining the optimal treatment course, energy strength, or configurations including pulse width, voltage, and frequency. A project to increase the TPRF voltage to 300 V is underway. Future research agenda for TPRF in addition to voltage amongst other things also needs to include current, electrode placement relative to pain, number of treatments, and gap between treatments, further comparative treatment with optimized conventional TENS and other common treatments.

## 14. Conclusion

When two transcutaneous stimulation techniques are used in chronic shoulder tendonitis pain, both TPRF and TENS are safe and effective after treatment and at follow-ups for 3 months. The effects of TPRF are superior to TENS although this superiority diminishes over time.

## Figures and Tables

**Table 1:** Demographic data, ASA status, duration of illness, and CMS/PEG before treatment.

Patients	Group TPRF (*n*=25)	Group TENS (*n*=25)	Levene test		*t* test	*p* value
			*F*	Sig.	*T*	
Age (year)	65.52 + 11.11	64.32 + 8.69	3.000	0.090		
Sex (M/F)	6/19	10/15	5.406	0.024^*∗*^	1.206	0.234
Weight (kg)	61.36 + 8.29	62.56 + 8.81	0.798	0.376		
ASA status (I/II)	8/17	7/18	0.366	0.548		
Duration of illness (m)	18.04 + 1.99	16.56 + 2.98	2.254	0.140		
CMS before treatment	40.44 + 7.71	38.72 + 9.56	0.491	0.487		
PEG before treatment	59.13 + 9.47	56.06 + 13.65	3.834	0.056		

^*∗*^ *p* < 0.05. Data are presented as mean + standard deviation. TPRF = transcutaneous pulsed radiofrequency; TENS = transcutaneous electrical nerve stimulation; n = number of the patients; ASA = American Society of Anesthesiologists physical status; CMS = Constant–Murley Shoulder score; PEG = pain, enjoyment of life, and general activity.

**Table 2:** Independent *t*-test for CMS for Groups TPRF and TENS.

Time	Levene test		*t*-test		*η* ^2^	Observed power
	F	Sig.	*T*	*p* value		
Before treatment	0.491	0.487			0.010	0.105
After treatment	7.871	0.007^*∗∗*^	4.844	0.000^*∗∗∗*^	0.328	0.997
1 w follow-up	0.541	0.466			0.205	0.931
1 m follow-up	0.227	0.636			0.204	0.931
3 m follow-up	0.069	0.794			0.173	0.873

^*∗∗*^ *p* < 0.05; ^*∗∗∗*^*p* ≤ 0.001; w = week and m = month.

**Table 3:** PEG score differences: ANOVA and Scheffe's post hoc analysis.

Timing	ANOVA	Post hoc *F* = 4.62 sig 0.005^*∗∗∗*^ *ω*^2^ = 0.13 (observed power 0.88)	
After 1 course	11.80 + 8.88	Group diff	*p* value
1 week later	6.07 + 7.04	5.73 + 2.16	0.077
1 month later	5.27 + 7.68	6.53 + 2.16	0.032^*∗*^
3 months later	4.67 + 6.77	7.13 + 2.16	0.016^*∗*^

^*∗*^ *p* < 0.05 and ^*∗∗∗*^*p* ≤ 0.001.

## Data Availability

The data used to support the findings of this study are available from the corresponding author upon request.
